# Formation of Composite Coatings on Galvanized Steel from Organosilane Solutions Using Electrophoresis and Sol–Gel Technology

**DOI:** 10.3390/ma15072418

**Published:** 2022-03-25

**Authors:** Yuriy B. Makarychev, Natalia A. Gladkikh, Galina V. Redkina, Oleg Yu. Grafov, Ali D. Aliev, Yuriy I. Kuznetsov

**Affiliations:** Frumkin Institute of Physical Chemistry and Electrochemistry, Russian Academy of Sciences, Leninsky Prospect 31-4, 119071 Moscow, Russia; makarychev-1949@mail.ru (Y.B.M.); gvredkina@mail.ru (G.V.R.); grafov.oleg88@gmail.com (O.Y.G.); ali_aliev1948@mail.ru (A.D.A.); yukuzn@gmail.com (Y.I.K.)

**Keywords:** corrosion, galvanized steel, phosphonates, organosilanes, sol–gel coatings, electrophoresis

## Abstract

New hybrid composite coatings (HCCs) on hot-dip galvanized steel (HDGS) were obtained using electrophoresis (cathodic polarization (CP)) and sol–gel technology. For this purpose, a technique for the preparation of a cationic precursor based on TiCl_4_ and aminopropyltriethoxysilane was developed. Electrophoresis of the charged particles of the precursor and organosilanes promotes the production of denser sol–gel coatings with improved adhesion. Using scanning electron microscopy (SEM), X-ray photoelectron spectroscopy (XPS) and electrochemical impedance spectroscopy (EIS) methods, the formation mechanism and protective properties of HCC on galvanized steel were investigated.

## 1. Introduction

Zinc is an amphoteric metal that has low resistance in alkaline and acidic solutions, especially when in contact with other metals. To protect zinc or zinc coatings on steel from corrosion, adsorption-type corrosion inhibitors are the most effective. Among organic zinc corrosion inhibitors in aqueous solutions, carboxylates [1,2,3,4], azoles and their substituted forms [5,6,7,8,9], molybdates [10,11], alkylphosphonic acids [11,12,13,14,15,16,17,18] and organosilanes [19,20,21] are currently known. Their inhibitory effect is often explained by the formation of a thin protective layer on the metal surface due to chemisorption or the formation of complex compounds with Zn^2+^ cations [5,11].

Recently, the modification of metal surfaces with composite coatings from aquatic inorganic–organic hybrid sols by sol–gel method has been of considerable interest. The sol–gel method is the process of gel formation through the sol stage [22,23,24,25,26]. Sols can be obtained from polymer (precursor) molecules capable of forming metal oxide nanoparticles dispersed in a liquid medium in the form of a three-dimensional grid (alkoxide gels) as a result of hydrolysis. Easily hydrolyzable compounds such as silicon alkoxides Si(OR)_4_ and a number of metals (Zr, Ti, Al, Sn) can be used as precursors. Sol nanoparticles are usually obtained by hydrolysis of Si tetraethoxysilane, Si(OC_2_H_5_)_4_∙(TEOS); zirconium N-propoxide, Zr(O(CH_2_)_2_CH_3_)_4_; or tetrachloride titanium, TiCl_4_. The process of forming gels from TEOS is described by the following equations [27,28]:

Si(OC_2_H_5_)_4_ + 4H_2_O → Si(OH)_4_ + 4C_2_H_5_OH (1)

Si(OH)_4_ → SiO_2_ + 2H_2_O (2)

The reaction rates (1) and (2) strongly depend on the pH and the amounts of added water and ethanol [29]. At pH < 7, the reaction (1) is quickly completed with the heating of the initial mixture, but the reaction (2) may take days or even months to complete. The result is a soft gel resembling gelatin. The gel is applied to the metal surface by dipping; then, it is dehydrated by air drying and annealing in an oven at high temperature. 

The inclusion of trialkoxysilanes in the composition of TEOS leads to the formation of new coatings, the so-called ormosils (ormosil—organically modified silica). The hydrolyzed particles of TEOS and silanols form composite coatings on the metal surface with high protective properties. Ormosil technologies are widely used for corrosion protection of aluminum alloys [30,31,32,33,34,35]. However, they are rarely used for zinc or zinc coatings [33,34,36] due to the use of acid and alkaline catalysts in them, which dissolve the zinc coating. Polymerization of organosilanes on the zinc surface can be accelerated due to various physical factors causing activation of the monomer near the surface. Such factors include the action of special radical initiators, ionizing radiation and electrophoresis [37,38]. Thus, in [39], it was found that the cathodic polarization of galvanized steel in a solution of bis-1,2-triethoxysilethane accelerates the formation of siloxane structures and denser polymer-like coatings. The formation of polymer-like coatings on the surface of metals using electrophoresis is based on the deposition of charged particles on the surface of electrodes due to the course of electrochemical and chemical reactions [38]. Organosilanes in which a vinyl group is present can acquire a positive charge with the help of acid catalysts by reaction:

HA + CH_2_ = CH − R → CH_3_ − CH_2_R^+^ + A^−^ (3)

Organosilanes containing an amino group have a positive charge on the nitrogen atom as a result of its protonation in acidic aqueous solutions:

NH_2_ − CH_2_ − R + H_2_O → [NH_3_ − CH_2_ − RH]^+^ + OH^−^ (4)

When the cathode potential is shifted, positively charged organosilane particles accumulate on the electrode surface, and the cathode space is alkalinized; in addition, the coating is dehydrated as a result of electrophoresis and electrolysis of water. A high concentration of organosilanes and alkaline catalysis leads to intensive polymerization on the metal surface. The combination of electrophoresis and ormosil technologies contributes to the formation of qualitatively new composite coatings.

In this paper, the features of adsorption and polymerization on the zinc surface of vinyltrimethoxysilane (VTMS) and aminopropyltriethoxysilane (AS) with precursors based on titanium tetrachloride will be investigated. These compounds belong to various types of organosilanes containing amine and vinyl groups of atoms in the composition of a hydrocarbon radical. A distinctive feature of these groups of atoms is that amino-containing organosilanes are bases and accelerate the polymerization process due to alkaline catalysis, and during the hydrolysis of VTMS, acidification of the solution occurs and polymerization is accelerated due to acid catalysis. In an acidic environment, AS and VTMS molecules carry a positive charge, which is important for the polymerization of organosilanes during cataphoresis. In this regard, it is of interest to apply hybrid technologies for applying composite coatings to HDGS, as well as to explore the possibility of using tetravalent titanium alkosilanes (TiRSis) as a polymerization precursor. 

Therefore, the purpose of this work is to use XPS and SEM to investigate the mechanism of formation and physicochemical properties of hybrid composite coatings obtained by electrophoresis and sol–gel technologies, to develop a method of obtaining and testing the effectiveness of a cationic precursor based on tetrachloride titanium and organosilanes, and to investigate the protective properties of the hybrid coatings using electrochemical methods of research and corrosion tests.

## 2. Experimental Section

### 2.1. Materials

In this research, hot-dip galvanized steel samples (Gust. Alberts GmbH & Co. KG, Herscheid, Germany) with dimensions of 100 mm × 60 mm × 0.8 mm were used. The thickness of the zinc coating ranged from 7 to 10 μm. Ultrasonic treatment for 15 min in acetone was used as a preliminary degreasing. Following, the samples were alkaline-treated in 1 M KOH solution for 5 min at T = 373 K. The purpose of this treatment was to provide alkaline etching and chemical activation of the surface.

In the preparation of working solutions, the following chemicals were used: vinyltrimethoxysilane (VTMS) H_2_C = CH − Si(−OCH_3_)_3_ (Witco Co., Geneva, Switzerland); aminopropyltriethoxysilane (AS) NH_2_ − (CH_2_)_3_Si(OC_2_H_5_)_3_ (Witco Co., Geneva, Switzerland); 2-hydroxy-1,2,3-propantricarboxylic acid (HPCA) (HOOCCH_2_)_2_ − C(OH)COOH (LenReactiv, Saint Petersburg, Russia); 1-hydroxyethane-1,1-diphosphonic acid (HEDP) CH_2_C(OH) − (PO(OH)_2_)_2_ (Ruschim, Moscow, Russia); tetrachloride titanium TiCl_4_ (Ruschim, Moscow, Russia); TiRSi complex Ti(NH_2_−(CH_2_)_3_−Si(OC_2_H_5_)_3_)_n_Cl_z_ (obtained in this work); acrylic–styrene dispersion of HomaCryl (Homa, Moscow, Russia); octadecylphosphonic acid (ODPA) CH_3_(CH_2_)_17_PO(OH)_2_ (Alfa Aesar, Haverhill, MA, USA). 

### 2.2. Obtaining HCCs on HDGS

The formation of HCCs on HDGS was carried out by four methods:

- Method A. An experimental solution (pH 4.5) consisting of VTMS, HEDP and HPCA in molar ratio 0.1/0.05/0.05 was kept for 24 h for a more complete hydrolysis of VTMS. Then, HDGS samples were immersed in it and kept for 20 min without cathodic polarization and with the cathodic potential superimposed at a current density of 10 mA/cm^2^. After exposure, the samples were dried in air at T= 333 K for 2 h and then subjected to heat treatment at T = 423 K for 20 min.
- Method B. For the preparation of solutions, TiRSi solutions in ethanol with water additives were used in the ratio EtOH:TiRSi:H_2_O 90:4:6% (*V*/*V*). The prepared mixture was kept for a day for the hydrolysis of TiRSi molecules. Before applying HCC on HDGS, 1 M KOH solution was added to the experimental mixture with stirring until an intense gel was formed. After exposure of the sample for 20 min, they were removed from the solution vertically with a lifting speed of 20 mm/min. Then, the samples were dried in air at T = 333 K for 2 h and subjected to heat treatment at T = 423 K for 20 min.
- Method C. VTMS (4% vol.) was added to the experimental water–alcohol solution (method B). The prepared mixture was kept for a day. After 1 M KOH addition and gel formation, the samples were kept in solution for 20 min without cathodic polarization and with cathodic potential superimposed at a current density of 10 mA/cm^2^. Then, the samples were dried in air at T = 333 K for 2 h and subjected to heat treatment at T = 423 K for 20 min.
- Method D. Acrylic–styrene dispersion of HomaCryl was used as a hydrophobic agent for impregnation of the obtained HCC and octadecylphosphonic acid (ODPA) CH_3_(CH_2_)_17_PO(OH)_2_. HDGS samples with HCC were briefly immersed in HomaCryl dispersion and then dried at T = 333 K for 60 min. ODPA was used as a 1.0 mM solution in ethyl alcohol. Impregnation of the HCC in ODPA solution was carried out at 313 K and constant stirring on a stirrer at a speed of 250 rpm for 2 h. After treatment in an alkylphosphonate solution, the samples were dried in a cabinet at 333 K for 60 min.

### 2.3. Characteristics of the HDGS Surface with HCC

#### 2.3.1. X-ray Photoelectron Spectroscopy (XPS)

Chemical analysis of the surface layers was performed by using an Omicron ESCA X-ray photoelectron spectrometer (Omicron Vakuumphysik GmbH, Taunusstein, Germany). An aluminum anode with a power of 250 watts was used. The transmittance energy amounted to 20 eV. The binding energy of the electrons escaping from the inner shells of the atoms was calibrated by the XPS peak of the C1s electrons, the binding energy of which has been accepted as 285.0 eV. The background was subtracted from the obtained spectra by the Shirley method [40]. The thickness of the protective layers was calculated using Multiquant 2.0 software (AB Sciex LLC, Framingham, MA, USA) [41].

#### 2.3.2. Scanning Electron Microscopy (SEM)

SEM images were obtained using a JSM U3 scanning electron microscope (Jeol, Tokyo, Japan). Analysis of the elements was performed with a scanning electron microscope equipped with a WinEDS energy-dispersive X-ray (EDX) analyzer (Eumex Instrumentebau GmbH, Heidenrod, Germany). The electron-beam intensity was 15 keV. The thickness of HCC was calculated using the GMRFilm software program. 

#### 2.3.3. Adhesion Measurement

**Figure 1:**
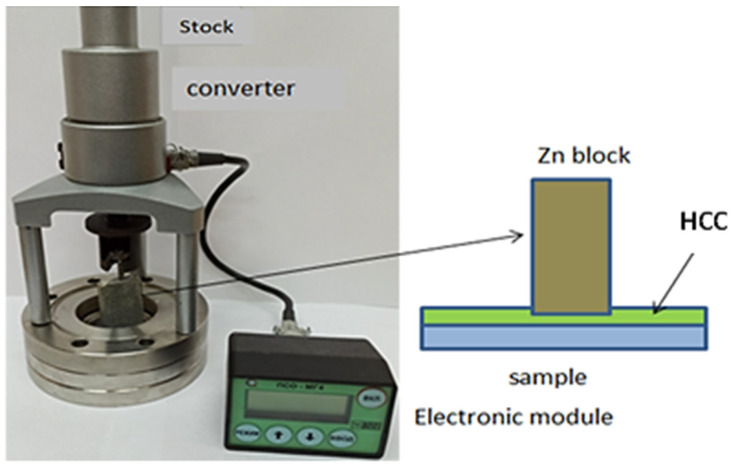
PSO-MG4 corrosion meter.

The tests were carried out using an electronic adhesion meter PSO-MG4 (Stroypribor, Chelyabinsk, Russia), as shown in Figure 1. 

The device used the pull-off method to remove HCC from the HDGS. The surfaces of Zn block and HDGS samples were coated with composite coatings. Then, after air drying at T = 333 K for 10 min, the samples were brought into contact and subjected to heat treatment for 60 min at T = 423 K. After sintering, adhesion was measured by placing the HDGS sample between two flanges, and the zinc block was attached to a movable rod. The contact area of the test specimens was 4 cm^2^. When the force transducer is loaded, the strain gauge transducer generates an electrical signal that changes in proportion to the applied load. The breakaway torque is controlled by an electronic module.

### 2.4. Investigation of the Protective Properties of HCC Formed on HDGS

#### 2.4.1. Wettability Measurements

The hydrophobic properties of the HCC were evaluated by the measurement of the static marginal angle (Θ*_s_*) of a drop of distilled water. Images of the water drop on the test surface were obtained on a laboratory device with a DCM 300 integrated camera (Hangzhou Huaxin IC Technology Inc., Zhejiang, China). To receive reliable information about the wettability of the surface, the measurements were made no later than 5 ÷ 10 s after setting the drop by at least 10 points on the surface. Drop volume in each experiment was 3 ÷ 5 µL. The values (Θ*_s_*) were determined by processing the obtained images in a graphic editor. The standard deviation was 1.0 ÷ 2.0°.

#### 2.4.2. Electrochemical Impedance Spectroscopy (EIS) 

EIS measurements were performed using Solartron’s Model 1250 potentiostat/galvanostat (Test Equipment Center Inc., Gainesville, GA, USA) and 1286 Frequency Response Analyzer (Test Equipment Center Inc., Gainesville, GA, USA). Tests were conducted in a three-electrode cell: silver chloride electrode was used as a reference electrode, platinum plate with a working area of 1 cm^2^ was used as an auxiliary electrode, and hot-dip galvanized steel samples were used as working electrodes (Section 2.1). A chloride-buffered solution of the following composition was used as the working solution: 0.4 M H_3_BO_3_ + 0.1 M Na_2_B_4_O_7_ + 0.01 M NaCl pH 6.7. Frequency scanning was performed by means of a sinusoidal wave perturbation with an amplitude of ±5 mV at the corrosion potential. The range of frequencies used ranged from 100 kHz to 1 mHz. Five impedance sampling points were recorded. The impedance data were analyzed using ZView software (Scribner Associates Inc., Southern Pines, NC, USA).

#### 2.4.3. Corrosion Testing

Corrosion tests of HDGS with coatings were carried out in a salt mist chamber (SMC), according to ISO 9227. HDGS samples with an area of S = 12 cm^2^ were placed in a Weiss WT 450 SMC (Weiss Umwelttechnik, Reiskirchen, Germany) after application, where a 5.0% NaCl solution (pH 6.5 = 7.2) was sprayed. The tests were carried out at 308 K and 95 ÷ 100% relative humidity.

## 3. Experimental Results

### 3.1. Results of XPS Investigations of Polymer-like Coatings

#### 3.1.1. Polymer-like Coatings Obtained by HCC on HDGS from Aqueous Solutions of Organosilanes by Method A

Investigations of the formation of polymer-like coatings on steel conducted by us earlier [34,35,36] showed that the most effective compositions are those that include organosilane VTMS, HEDP and BTA. In this mixture, the hydrolyzed form of the VTMS molecule is a monomer in polycondensation reactions, while HEDP and BTA are corrosion inhibitors. BTA cannot be a useful additive for zinc because it is not a corrosion inhibitor for this metal and has only one functional atom group capable of polycondensation. It is known [36] that monofunctional additives are blockers of branched polymer chains, so copolymers with a large number of functional groups are usually used. Therefore, instead of BTA, we chose HPCA, which has four functional groups capable of participating in polycondensation reactions. The choice of HEDP as a component of the composition is due to the fact that phosphonic acids and their salts are capable of forming complex insoluble compounds on the surface of metals [6,7] and are also copolymers in polymerization reactions.

The study of polymer layers on zinc obtained from aqueous solutions of organosilanes was carried out using XPS. After HCCs were obtained, HDGS samples were washed in running and distilled water using ultrasound for 5 min to remove electrolyte residues. Table 1 shows the chemical composition and thickness of the surface layers on HDGS after various processing methods.

**Figure 2:**
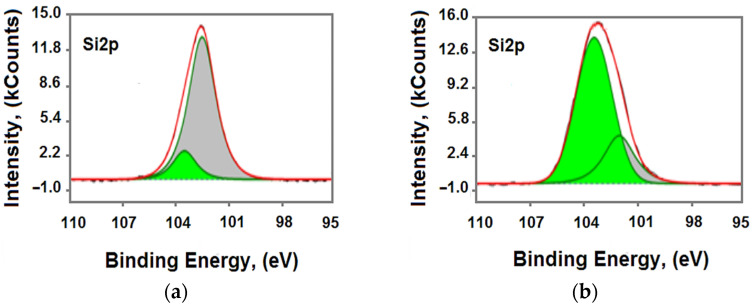
X-ray photoelectron spectra of Si2p surface of HDGS samples after exposure in 0.1 M VTMS + 0.05 M HEDP + 0.05 M HPCA solution for 20 min: (**a**) without CP; (**b**) with CP with current density i = 10 mA/cm^2^.

The results of these studies have shown that cathodic polarization significantly accelerates the growth of polymer layers on the HDGS. With cathodic polarization, the thickness of the polymer layers is much greater, and organosilicon structures predominate in the chemical composition. Analysis of the XPS spectra showed that silanol and siloxane groups of atoms are present in these structures. After the Si2p spectrum is decomposed into components (Figure 2), two peaks with E_b_ = 102.4 eV and E_b_ = 103.7 eV can be distinguished, which belong to the silanol and siloxane groups of atoms, respectively [35].

An increase in the number of siloxane structures in the coating indicates that cathodic polarization accelerates the polymerization process of the coating. It is obvious that there are optimal current densities that ensure the maximum growth rate of polymer films. In our case, i_c_ = 10 mA/cm^2^ is empirically chosen. At a lower i_c_, the concentration of the mixture components in the near-electrode space is small, and at a higher i_c_, the formation of the film is hindered by the intense release of hydrogen.

#### 3.1.2. Polymer-like Coatings Obtained by HCC on HDGS from Aqueous-Alcohol Solutions of Organosilanes by Methods B and C

**Scheme 1:**
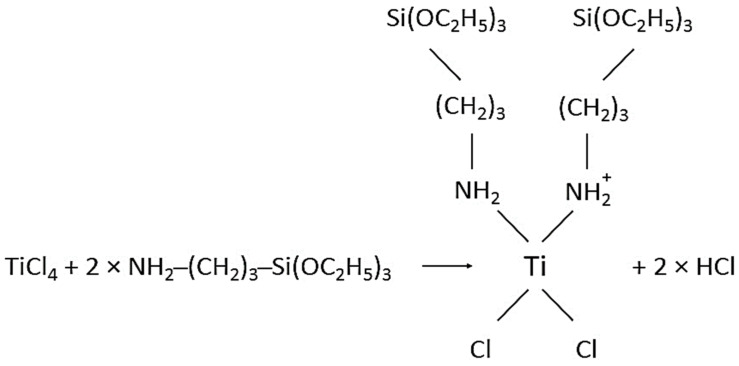
The amino group substitution reaction.

According to the literature and our data [33,34], the thickness of polymer layers obtained from organosilane mixtures without special initiators does not exceed several fractions of a micron. To protect against atmospheric corrosion in conditions of high aggressiveness of the environment, such a coating thickness is not enough. In addition, without cathodic bias, it is difficult to control the thickness and good adhesion of the coating. In this regard, the solution to the problem may be the use of cataphoresis in ormosil technology. To do this, it is necessary to have positively charged particles in the precursor and silanol compositions. Hydrolyzed TEOS molecules and zirconium N-propoxide are electroneutral and are inactive in electrophoresis. In this regard, TiCl_4_ was chosen for the synthesis of positively charged sol–gel particles. This compound has an extremely high chemical activity and easily enters into reactions of nucleophilic substitution of chloride ions with proton-containing molecules. Together with AS containing the terminal amino group, the substitution reaction proceeds according to Scheme 1.

**Figure 3:**
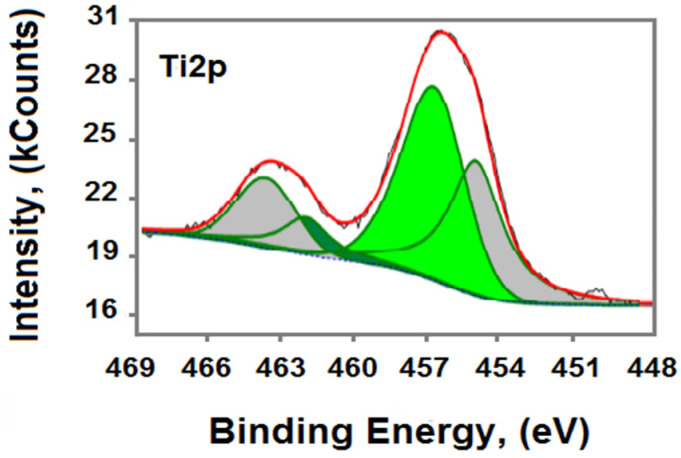
X-ray photoelectron spectra of Ti2p adsorbed on the HDGS surface of the TiRSi molecule.

The formation of the Ti(NH_2_−(CH_2_)_3_−Si(OC_2_H_5_)_3_)_n_Cl_z_ (TiRSi) complex, with a given number of substituted radicals, can be achieved by mixing solutions of TiCl_4_ and AS with the necessary molar ratio of the initial components. When mixing the solution, its strong heating occurs, as does a color change. This indicates the course of a chemical reaction. When a complete replacement of chlorine atoms with AS radicals is required, the solution heat is completely stopped. Studies of the chemical composition of TiRSi were carried out. To do this, HDGS samples were immersed in an anhydrous TiRSi solution for 10 min. After exposure, the samples were kept in air at T = 333 K until the liquid phase of TiRSi was completely removed. The purple oxide salt precipitate that remained on the surface was analyzed using XPS. On the Ti2p spectrum, there is a peak with an energy of 457.4 eV, which refers to TiRSi molecules, and a peak with an energy of 458.7 eV, which refers to TiCl_4_ molecules or products of their hydrolysis, TiO_2_ (Figure 3). 

**Figure 4:**
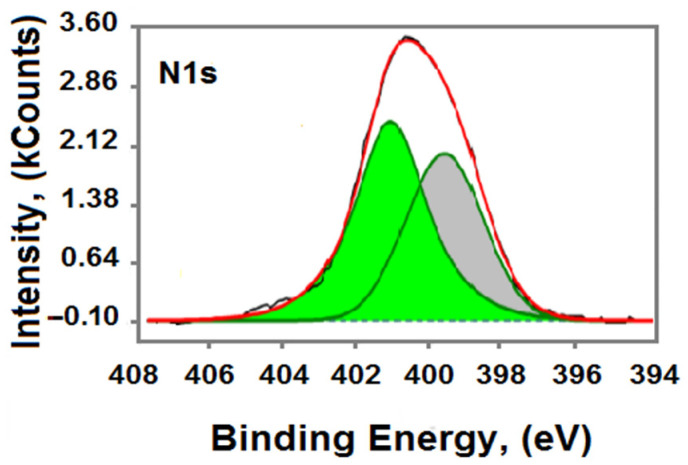
The X-ray photoelectron spectra of N1s adsorbed on the surface of the HDGS TiRSi molecule.

On the N1s spectrum (Figure 4), two peaks with energies of 399.8 eV and 401.7 eV, belonging to neutral and protonated nitrogen atoms, respectively, are observed. The relative amounts of nitrogen and titanium in these spectra are close to two, which corresponds to the TiRSi complex obtained by the reaction in Scheme 1.

The TiRSi complex is stable in acidic and neutral environments, has a positive charge on a protonated nitrogen atom and has three functional ethoxy groups of atoms capable of polymerization reactions. However, in alkaline solutions, rapid hydrolysis of complexes occurs with the formation of TiO_2_ sol–gel and organosilanol TiRSi molecules. These features of TiRSi complex compounds can be used in the development of hybrid technologies for obtaining protective coatings using electrophoresis and ormosil methods. In this case, TiRSi molecules served as a promoter during the formation of sol–gel inorganic–organic hybrid coatings. 

**Figure 5:**
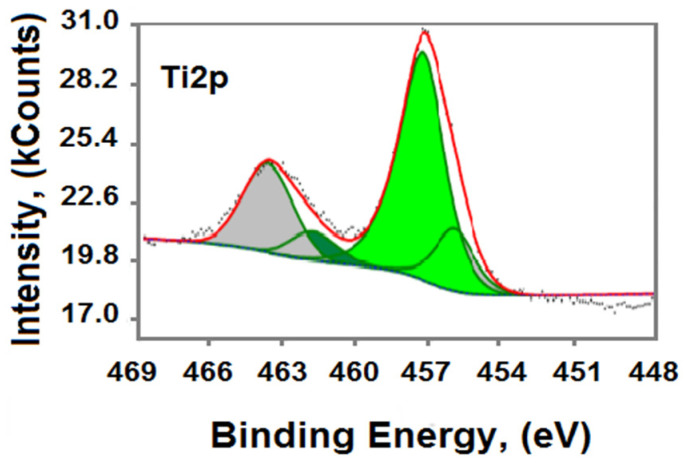
X-ray photoelectron spectra of Ti2p HDGS samples after exposure in a modified TiRSi solution for 20 min.

After processing the samples by method B, an HCC is formed on the HDGS surface, consisting of a polymer-like matrix with ultradisperse TiO_2_ particles included in it. XPS analysis of the HCC surface showed that these particles have a complex chemical composition. Figure 5 shows the spectrum of Ti2p after exposure to HDGS in a modified TiRSi solution. It can be seen that titanium compounds in the form of oxides, chlorides and TiRSi complexes are present in the composition of the HCC.

**Scheme 2:**
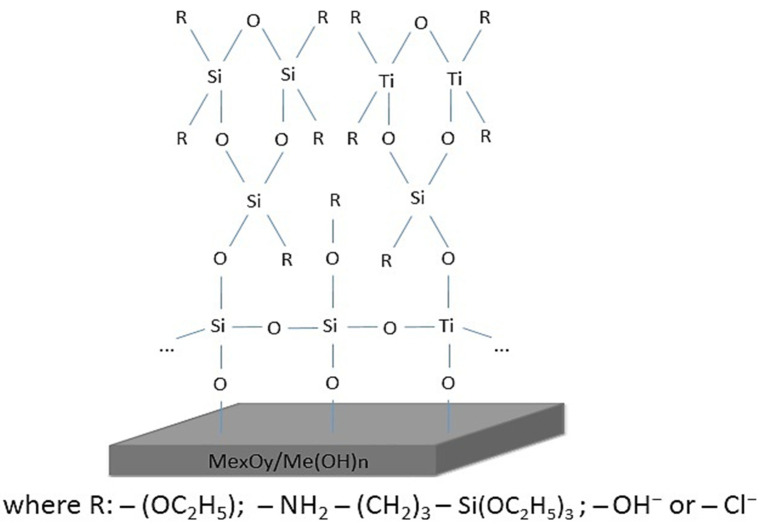
The general scheme of the formation of polymer layers on the HDGS surface.

The formation of polymer coating in introduced solutions of organosilanes with TiRSi precursor additives can be represented as Scheme 2.

In the layer adjacent to the metal surface, a polymer two-dimensional structure is formed from molecules of organosilanes and a precursor that is chemically bound to the metal and subsequent three-dimensional cyclic siloxanes and alkyltitanates. The ultradisperse TiO_2_ particles formed as a result of the hydrolysis of precursor molecules are embedded in the organic matrix of the HCC.

### 3.2. Results of SEM Investigations

**Figure 6:**
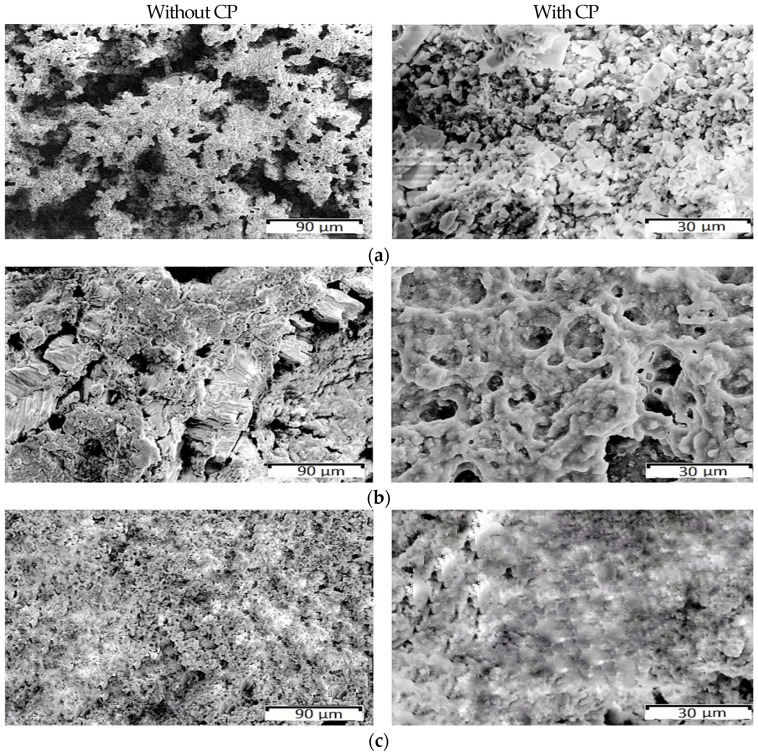
Electronic microphotography of the surface HCC after modification: (**a**) method A; (**b**) method B; (**c**) method C.

SEM investigations were performed on HDGS samples on which polymer-like coatings obtained by methods A, B and C without/with CP (Figure 6) were formed. According to Figure 6a, showing the polymer-like coating obtained by method A in the absence of CP, the sol–gel particles are deposited on the surface of the samples in the form of individual aggregates, weakly connected to each other and having a spongy structure. Under the influence of CP, the structure of the polymer-like coating becomes more homogeneous (Figure 6a). As shown in SEM images (Figure 6b), the structure of the electrophoretic coating (method B) of the HCC without CP is defective, exhibiting through pores and microcracks, and is similar to that obtained by method A in the presence of CP. In the presence of CP, the coating shows an absence of cracks and through pores. The densest structure of the polymer-like coating both without and with CP was registered in the samples made by method C (Figure 6c). The formation of a denser structure is probably due to the presence of oxides and complex compounds of titanium. This follows from the surface microanalysis data of the samples measured after the two coating methods, as shown in Table 2. The increase in the concentrations of nitrogen and titanium in the HCC seems to be due to the accumulation of the non-hydrolyzed form of TiRSi complexes. 

Table 2 shows the chemical composition of the HCCs obtained by SEM microanalysis.

From the data given in Table 2, it can be seen that with cathodic polarization, the proportion of the inorganic phase in the organic–inorganic coating increases. The compaction of HCC promotes the formation of three-dimensional siloxane structures with inclusions in the form of hydrolyzed TiRSi molecules chemically bound to silanol groups of atoms. These structures form the basis of an organic–inorganic matrix into which ultradisperse TiO_2_ particles are dispersed.

The mechanism of formation of such coatings lies in the fact that during cathodic polarization, positively charged particles of the mixture components accumulate at the surface of the electrode. At the same time, the near-electrode space is alkalized, which contributes to the hydrolysis of the TiRSi and organosilane complex. This is also facilitated by the viscous medium of the formed sol–gel and the presence of ultrafine TiO_2_ particles, which are the centers of coordination of organosilanol molecules and the development of three-dimensional polymer structures. The electrostatic attraction and electrophoresis of the components of the mixture, as well as the electrolysis of solvent molecules, contribute to the sealing of the coating. 

### 3.3. Investigation of Polymer-like Coatings Obtained by Method D on HDGS

**Figure 7:**
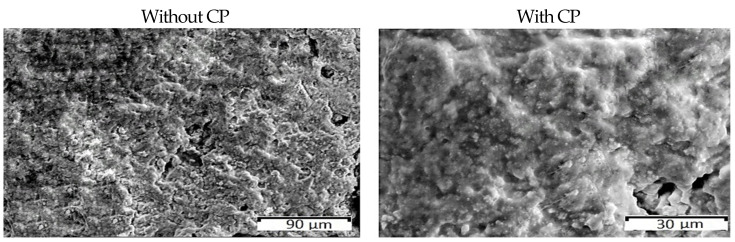
Electronic microphotography of the surface HCC after modified by method D.

Despite the composite coatings obtained by electrophoresis and sol–gel technology having a denser and more homogeneous structure, as can be observed in Figure 6c, there are still pores in the polymer-like coating. Their presence does not prevent the penetration of moisture and corrosive substances to the metal surface. In this regard, to improve the protective properties of the polymer-like coating, the samples were treated according to method D. Figure 7 illustrates SEM images of the coatings obtained by method D with/without CP. 

From the presented SEM images, it is obvious that the structures of polymer-like coatings obtained by method D are the least defective compared with the coatings shown in Figure 6. In this regard, it is most relevant to obtain information on the adhesion strength of the coatings obtained by method D. Studies of the adhesion of HCCs on HDGS after various technological operations were carried out. The results of the studies are presented in Table 3.

From the data shown in Table 3, it can be seen that with cathodic polarization, the adhesion force increases by 29%. Summarizing the obtained results, it can be assumed that the use of CP leads to the formation of a more homogeneous structure of the polymer-like coatings. By filling the pores with hydrophobic molecules of HomaCryl dispersion and ODPA, the HCC structure becomes denser, and the molecules are uniformly distributed over the volume and surface, which is confirmed by the results provided in Figure 7 and Table 3. The presence of ODPA molecules obtained by method D in the coating composition should result in improved corrosion resistance of HCCs on HDGS samples. Further investigations were conducted to analyze the protective properties of the coatings obtained by method D in the presence of CP.

#### 3.3.1. The Results of Wettability Measurements

As described above, the protective properties of the coatings obtained by method D were investigated. For all investigations, samples exposed to CP were applied. The following samples were tested: the sample shown in Figure 6c (blank), blank + HomaCryl and the sample prepared by method D (Figure 7, with CP). Since HomaCryl and ODPA were used as hydrophobizers for the coating surface, wettability investigations were conducted on these samples. The results of these investigations are described in Table 4. 

As can be observed in Table 4, the treatment of the sample by method B, although it does not allow obtaining a superhydrophobic coating, provides a high hydrophobization of the surface.

#### 3.3.2. The Results of EIS Measurements

**Figure 8:**
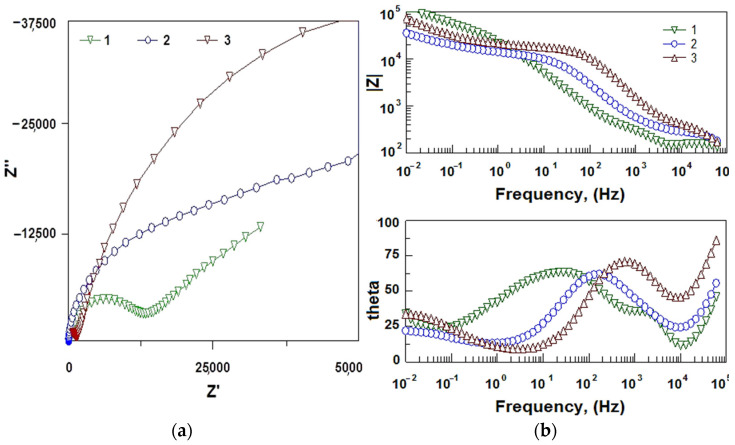
EIS diagrams: (**a**) Nyquist plot; (**b**) Bode plot obtained in a borate buffer solution containing 0.01 M NaCl on HDGS samples with HCC: 1, blank; 2, blank + HomaCryl; 3, method D.

Figure 8 illustrates the EIS results of the tested samples. The hodograph on the EIS diagram obtained on the blank sample has the form of a symmetrical semicircle in the high-frequency region and a rectilinear section at low frequencies less than 100 Hz. This type of hodograph is typical for porous samples with diffusion limitations and corresponds to the Warburg impedance. There are two clear maxima on the Bode graph related to two different mechanisms of charge transfer through the coating. The maximum at low frequencies is associated with electrochemical processes occurring at the metal–solution interface, and the maximum at high frequencies is associated with charge transfer through the coating.

**Figure 9:**
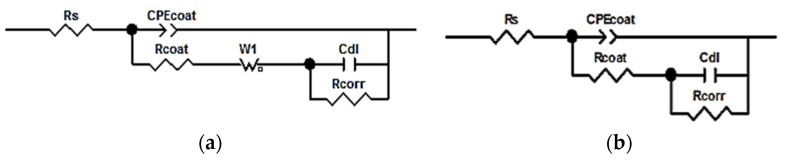
Equivalent impedance scheme for HDGS samples with HCC: (**a**) without impregnation; (**b**) with impregnation. Rs is the resistance of the electrolyte, CPE_coat_ is the element of the constant phase, R_coat_ is the ohmic resistance of the coating, C_dl_ is the capacitance of the double electric layer at the metal boundary/coating, R_corr_ is the resistance of electrochemical reaction and W1 is the Warburg impedance.

On the blank + HomaCryl sample, the porosity of the coating decreases, and the charge transfer resistance through the coating increases. The hodograph on the EIS diagram takes the form of a semicircle with a higher value and a linear beam at an angle of 45° at low frequencies. This type of hodograph is typical for porous samples with diffusion limitations and corresponds to the Warburg impedance, which we can observe in Figure 8. On the Bode graph, both maxima are transformed into one symmetrical maximum shifted to the high-frequency region of the hodograph. The disappearance of the Faraday impedance maximum indicates the termination of access of corrosive components to the metal surface. To calculate the EIS diagrams of the parameters, two equivalent schemes were used, shown in Figure 9 and Table 5.

From the data shown in Table 5, it can be seen that after the HCC is impregnated with HDGS hydrophobizers, the charge transfer resistance through the R_coat_ film increases significantly. This is primarily due to the diffusion limitations of the delivery of corrosive substances to the metal surface. Obviously, at the same time, the protective properties of the HCC increase.

#### 3.3.3. The Results of Corrosion Tests

**Figure 10:**
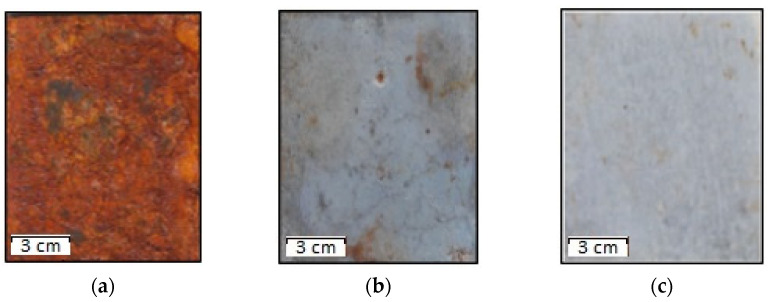
Surfaces of HDGS samples after 200 h of testing in SMC: (**a**) blank; (**b**) blank + HomaCryl; (**c**) method D.

To assess the anticorrosive efficiency of the HCC, corrosion tests of HDGS samples were carried out in a salt mist chamber. Figure 10 shows photos of the HDGS surface after 200 h in a salt mist atmosphere.

On the blank specimen, corrosion damages are detected over almost the whole surface. The protective properties of blank + HomaCryl are strengthened, although a few corrosion areas appear during the testing. At the same time, the sample treated by method D is almost completely protected against corrosion. Thus, the HCCs obtained on the HDGS samples with the help of ormosil technology and electrophoresis exhibit weak protective properties in the SMC atmosphere. At the same time, having high porosity and good adhesion to a metal substrate, they can be the basis for obtaining highly effective protective coatings after impregnation with hydrophobic agents.

## 4. Discussion

The conducted studies have shown significant advantages of HCCs on HDGS obtained using electrophoresis and ormosil technologies. With cathodic polarization of the electrode in the near-electrode layer of the solution, the number of positively charged precursor particles and organosilanes increases, while maintaining a low concentration of components and pH of the solution in the volume of the solution. This is necessary when it is required to maintain a stable state in solutions at abnormally high rates of hydrolysis of precursors and polycondensation of silanols. The initiation of hydrolysis and polycondensation processes near the electrode surface is associated with the accumulation of charged particles of reagents and alkaline catalysis as a result of the electrolysis of water molecules [30,31,32,33,37,39]. For zinc coatings, such additives can be derivatives of phosphonic and carboxylic acids, multifunctional organosilanes and azoles and their substituted forms [5,7,28,29,35].

With the help of XPS studies, it was found that cathodic polarization accelerates the polymerization of organosilanols. Coatings become denser due to their dehydration as a result of electroosmosis and electrolysis of water molecules. Compaction of the coating and dehydration contribute to the formation of siloxane bonds and polymerization processes.

The method of synthesis of a precursor based on TiCl_4_ and aminopropyltriethoxysilane proposed by us for the first time makes it possible to use the precursor in sol–gel technologies and obtain coatings with increased protective properties. The anticorrosive effectiveness of HCCs obtained by electrophoresis and ormosil technology with subsequent treatment with hydrophobizers (HomaCryl acrylic–styrene dispersion and ODPA alcohol solution) has been proven by the results of corrosion tests. The impregnation of such coatings makes it possible to achieve high hydrophobization of the surface, thereby significantly increasing their protective properties in an aggressive atmosphere [42]. The conducted research can be useful for various technologies for obtaining organic–inorganic HCCs for metal.

## 5. Conclusions

As the result of the investigation, the following conclusions can be made:

1. The application of electrophoresis and ormosil technology on the samples of hot-dip galvanized steel resulted in the creation of hybrid composite coatings (HCCs).
2. XPS and SEM analyses, as well as the results of adhesion tests, have shown that cathodic polarization (CP) accelerates the process of polymerization of HCCs. Using CP, it is feasible to control the thickness and adhesion strength. The optimum cathodic current density (i_c_ = 10 mA/cm^2^) providing maximum growth rate of polymer films, adhesion strength and thickness has been empirically determined.
3. The addition of TiCl4 and aminopropyltriethoxysilane to the sol–gel facilitates the formation of three-dimensional siloxane structures. These structures create the basis of a polymer-like matrix with ultradispersed TiO2 particles incorporated within it.
4. Subsequent treatment of HCCs with hydrophobizers (acrylic–styrene dispersion HomaCryl and an alcohol solution of ODPA) achieves high surface hydrophobization, therefore significantly increasing their protective properties in an aggressive atmosphere.

## Figures and Tables

**Table 1:** Chemical composition (measured by XPS) and thickness of surface layers (measured by SEM) on HDGS samples.

Conditions for Obtaining HCC	Si, at. %	Zn, at. %	P, at. %	C, at. %	O, at. %	h, nm
Without polarization	8.3	4.2	1.9	49.1	36.5	10 ± 2
Cathodic polarization (CP)	14.6	2.3	2.5	38.4	42.2	120 ± 10

**Table 2:** Chemical composition of HCCs.

Treatment	Si, at. %	Ti, at. %	N, at. %	Cl, at. %	C, at. %	O, at. %
Without CP	13.5	4.4	1.1	2.1	36.1	41.0
With CP	17.7	6.9	1.9	1.3	29.3	42.9

**Table 3:** HCC adhesion on HDGS samples prepared by method D.

Treatment	Adhesion Strength, MPa	Coating Thickness, µm
Without CP	1.25 ± 0.05	8 ± 0.5
With CP	1.76 ± 0.05	15 ± 0.5

**Table 4:** Photographs of drops of distilled water and the values of Θ_C_ (°) on HDGS samples with HCC.

Blank	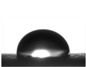 60 ± 1
Blank + HomaCryl	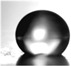 107 ± 3
Method D	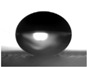 144 ± 3

**Table 5:** Parameters of the equivalent scheme for HDGS samples with HCC without and with impregnation with hydrophobizers.

Type of HCC	R_coat_, kOm × cm^−2^	R_corr_, kOm × cm^−2^	C_dl_, μF × cm^−2^	W_R_, kOm × cm^−2^
Blank	38.8	4.12	18.4	6.7
Blank + HomaCryl	182.6	15.7	4.7	–
Method D	263.2	32.4	1.12	–

## Data Availability

Not applicable.
